# An exploration of socioeconomic variation in lifestyle factors and adiposity in the Ontario Food Survey through structural equation modeling

**DOI:** 10.1186/1479-5868-4-8

**Published:** 2007-03-29

**Authors:** Heather Ward, Valerie Tarasuk, Rena Mendelson, Gail McKeown-Eyssen

**Affiliations:** 1 Department of Nutritional Sciences, University of Toronto, 150 College Street, Toronto, Ontario, M5S 3E2, Canada; 2 School of Nutrition, Ryerson University, 350 Victoria Street, Toronto, Ontario, M5B 2K3, Canada; 3 Department of Public Health Sciences, University of Toronto, 155 College Street, Toronto, Ontario, M5T 3M7, Canada

## Abstract

**Title:**

An exploration of socioeconomic variation in lifestyle factors and adiposity in the Ontario Food Survey through structural equation models.

**Background:**

Socioeconomic indicators have been inversely associated with overweight and obesity, with stronger associations observed among women. The objective of the present secondary analysis was to examine the relationships among socioeconomic measures and adiposity for men and women participating in the Ontario Food Survey (OFS), and to explore lifestyle factors as potential mediators of these associations.

**Methods:**

The cross-sectional 1997/98 OFS collected anthropometric measurements, a food frequency questionnaire, data on socio-demographics (age, sex, income, and education) and physical activity from 620 women and 467 men, ages 18 to 75. Based on the 2003 Health Canada guidelines, waist circumference and BMI values were used to derive least risk, increased risk, and high risk adiposity groups. Structural equation modeling was conducted to examine increased risk and high risk adiposity in relation to education and income, with leisure time physical activity, fruit and vegetable intake, and smoking status included as potential mediators of these associations.

**Results:**

The probability of high risk adiposity was directly associated with education (β-0.19, p < 0.05) and income (β-0.22, p < 0.05) for women, but not for men. Fruit and vegetable intake was a marginally significant mediator of the relationship between education and high risk adiposity for women. Increased risk adiposity was not associated with income or education for men or women.

**Conclusion:**

The socioeconomic context of adiposity continues to differ greatly between men and women. For women only in the OFS, fruit and vegetable intake contributed to the inverse association between education and high risk adiposity; however, additional explanatory factors are yet to be determined.

## Background

While it is popular to examine specific foods or sedentary activities as causes of rising obesity rates, it is likely that obesity is related to a complex set of sociodemographic and behaviourally based variables that influence overall lifestyle. Previous studies have found a lower prevalence of obesity among adults with higher levels of education [1-7], with stronger evidence of this association for women than men. The relationship between income level and obesity has also been found to vary by sex, and has been less consistent in its direction than education [1,5,8,9]. Lifestyle behaviours that have been associated with obesity include leisure time physical activity (LTPA) [10-13], fruit and vegetable intake [5,14], and smoking [10,12,15-17]. These factors have also demonstrated socioeconomic variation in several populations: higher levels of income or education have been associated with higher levels of leisure time physical activity [1,2,18-20] and higher fruit and vegetable intake [2,8,14,21-28] compared to lower income and education groups. Based on these studies, it is reasonable to suggest that some of the inverse association between socioeconomic indicators and adiposity may be occurring indirectly (i.e. mediated) through dietary factors and activity.

The contribution of smoking to the relationship between socioeconomic measures and adiposity is less clear. Higher levels of smoking have been observed among adults with lower income and education [1,2,19,20,29,30], however, evidence of lower BMI among current smokers suggests that this behaviour ought to reduce the likelihood of obesity rather than increase it [10,16,31]. Similarly, a previous history of smoking has been associated with increased adiposity, yet there is evidence to indicate that the socioeconomic groups most likely to have quit smoking are those with high income or high education [32,33]. A simultaneous examination of the indirect contributions of smoking, fruit and vegetable intake, and LTPA to the association between SES and adiposity could clarify some of the contradictory associations observed in the literature.

Associations among socioeconomic indicators, lifestyle factors, and adiposity are often examined through multivariate regression analyses; this approach provides insight into independent associations among pairs of variables (e.g. education and LTPA), but does not allow for direct and indirect effects to be explored as there is typically only one dependent variable under investigation. Structural equation modeling (SEM), a regression-based technique that incorporates factor analysis for the creation of latent variables, enables the simultaneous estimation of direct and indirect pathways in models with multiple dependent variables [34]. Examples of previous SEM analyses include a study of fruit and vegetable intake in relation to personal, behavioural, and socio-economic factors [35], and an examination of stress as a mediator of the association between primary determinants of health (socioeconomic and demographic factors) and health status [36].

The objective of the present SEM analysis was to examine the direct and indirect associations among socioeconomic indicators, lifestyle factors, and adiposity for adults who participated in the Ontario Food Survey (OFS). This data set was appropriate for such an exploration due to the breadth of measures included in the survey. Waist circumference measurements were included in the OFS in recognition of the health risks associated with central adiposity [37-39]. The adiposity categories for the present analysis were based on the Canadian Guidelines for Body Weight Classification [40], in accordance with the World Health Organization cut points [41]. The present analyses sought to evaluate whether associations of income and education with lifestyle factors and adiposity differed according to degree of adiposity.

## Methods

### Sampling and Recruitment

The OFS was conducted between June of 1997 and September of 1998. A full description of the survey method had been published previously [42]. Participants were drawn from the Ontario Health Insurance Program database using a stratified multistage probability design, and were contacted by letter and follow-up phone call. In-home interviews were conducted with adults between the ages of 18 and 74. Completed surveys were obtained from 707 women and 480 men. Thirty-six percent of those who were contacted gave oral consent during the telephone recruitment; however, with a high number of participants not reached, the overall response rate was twenty-nine percent. The study protocol was approved by the Ethics Research Board at the University of Toronto and Ryerson University.

### Anthropometrics

Participants were weighed to the nearest 0.5 kg on a calibrated mechanical dial scale. Height and waist circumference (WC) were measured with a measuring tape to the nearest 0.1 cm. BMI was defined as weight (kg)/height (m^2^). In the Canadian Guidelines, additional categories provide distinction between 'very high risk' adults (BMI of 35–39.9) and 'extremely high risk' adults (BMI > 40) regardless of WC. However, in the interest of avoiding insufficient sample size within adiposity groups, adults with a BMI of 30 or greater were included in the high risk adiposity category, regardless of WC (table 1).

**Table 1:** Adiposity Risk Categories †

	Least risk	Increased risk	High risk
Men	BMI 18.5–24.9 WC < 102 cm	BMI 18.5–24.9, WC > 102 cm BMI 25–29.9, WC < 102 cm	BMI 25–29.9, WC > 102 cm BMI > 30, any WC
Women	BMI 18.5–24.9 WC < 88 cm	BMI 18.5–24.9, WC > 88 cm BMI 25–29.9, WC < 88 cm	BMI 25–29.9, WC > 88 cm BMI > 30, any WC

† Adapted from the Health Canada Canadian Guidelines for Body Weight Classification in Adults

### Demographic and lifestyle characteristics

An interviewer-administered questionnaire was used to collect data on participants' age, education, and household income (before tax). The income categories were based on multiples of the Statistics Canada household low-income cutoff, which varies by region of residence and household size [43]. A lifestyle questionnaire addressed the frequency and duration of vigorous leisure-time physical activity (LTPA); these data were used to categorize participants as having high, medium, low, or inactive LTPA levels. The Canadian Society for Exercise Physiology's recommendation of 20–30 minutes of vigorous activity on four days a week was used to approximate the medium LTPA category definition[44] The high and low LTPA categories were then based on frequency/duration combinations that were relatively higher and lower than the medium level. Participants were asked if they were current, former, or never smokers.

### Fruit and vegetable intake

Interviewers administered a food frequency questionnaire (FFQ) designed by Health Canada for all of the provincial surveys that addressed the serving size and frequency of intake of 17 specific fruits and vegetables (including juices) during the previous month [45]. There were also two open-ended questions on the intake of 'other' fruits and vegetables that enabled respondents to report items not included in the FFQ.

### Structural equation modeling

The interrelationships among socioeconomic indicators, lifestyle factors, and adiposity in the OFS were examined simultaneously through SEM analyses, conducted with MPlus version 4. The present analyses included the 620 women and 467 men for whom height and weight data were available, and who were not underweight (BMI < 18.5). The weighted least squares method (WLSM) was used as the estimator in the present analyses. All models were evaluated based on their fit statistics, which are a measure of how well the covariance among variables as specified in the model corresponds to the observed covariance in the data [34]. The model fit statistics examined in the present analyses were the chi-square, root mean square error of approximation (RMSEA) and comparative fit index (CFI); acceptable fit between the proposed model and the data set was indicated by RMSEA values of 0.08 or less and CFI close to 0.95 [46]. In order to avoid listwise deletion of participants with missing data points, the maximum likelihood estimator (ML) function of MPlus was used [47]. The ML approach estimates a likelihood function for each individual based on the variables that are present so that all the available data are used.

Relationships between variables were measured with regression coefficients (β), with significance noted at p values below 0.05 [48], and the presented model parameters have been standardized. Indirect associations were calculated as the product of the standardized regression coefficients of the pathway components [34]; this product was then divided by the product of the standard errors from the pathway components to assess the significance of the indirect effects.

### Model modification and presentation

**Figure 1:**
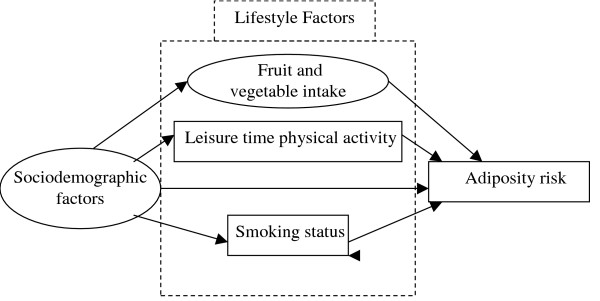
Heuristic model for SEM analyses.

A simplified version of the model to be tested is presented in figure 1. Adiposity groups were examined separately with least risk serving as the reference group, and all analyses were stratified by sex. Income and education were also examined separately, as the literature suggests these socioeconomic indicators have different associations with adiposity. Given the wide age range of participants in the OFS, age was included as a covariate in all regressions. Preliminary analyses revealed that the usual intake of both fruits and vegetables was related to adiposity classification, therefore, a latent variable combining fruit and vegetable intake was derived for the structural equation models. The three smoking categories were entered into the model as two dichotomous dummy variables, current and former, with the never smoked group serving as the reference. Correlations from fruit and vegetable intake to LTPA and to the smoking dummy variables were included as these associations have been previously observed in the literature [14,30]. The initial model did not yield an acceptable fit (Appendix Table 2). Data from residual output indicated that model fit would be improved if the 'current smoker' and 'former smoker' dummy variables were allowed to covary. The correlation between the dummy variables was likely due to their shared reference group (never smokers), and the inclusion of this correlation yielded acceptable fit. Given that the primary focus of the present analyses is the identification of factors directly and indirectly related to adiposity, correlation values between lifestyle factors are not presented.

**Table 2:** Appendix, Model fit statistics for initial model †

Model	Chi-square, p-value	CFI	RMSEA
**Men**			
High risk adiposity, income, LTPA, fruit and vegetable, age, current and former smoking	60.55, 0.000	0.58	0.216
High risk adiposity, education, LTPA, fruit and vegetable, age, current and former smoking	137.14, 0.00	0.27	0.312
Increased risk adiposity, income, LTPA, fruit and vegetable, age, current and former smoking	47.24, 0.000	0.59	0.165
Increased risk adiposity, education, LTPA, fruit and vegetable, age, current and former smoking	66.83, 0.000	0.55	0.206
			
**Women**			
High risk adiposity, income, LTPA, fruit and vegetable, age, current and former smoking	96.41, 0.000	0.56	0.218
High risk adiposity, education, LTPA, fruit and vegetable, age, current and former smoking	108.17, 0.000	0.59	0.191
Increased risk adiposity, income, LTPA, fruit and vegetable, age, current and former smoking	63.59, 0.000	0.53	0.169
Increased risk adiposity, education, LTPA, fruit and vegetable, age, current and former smoking	73.05, 0.000	0.55	0.169

† differs from final model shown in results section only insofar as current and former smoking variables are not co-varied in the initial model

## Results

The distributions of sociodemographic characteristics, adiposity risk, and lifestyle behaviours of the OFS participants are presented in tables 3 and 4.

**Table 3:** Distribution of socio-demographic characteristics and adiposity in the OFS

Variable		Men N = 467		Women N = 620	
		n	%*	n	%*

Age category	18 – 34	96	21	157	25
	35 – 49	117	25	180	29
	50 – 64	134	29	178	29
	54 – 75	120	26	105	17
					
Income	Missing	54	12	105	17
	Very low	40	9	95	15
	Low	87	19	150	24
	Middle	106	23	110	18
	High	180	39	160	26
					
Education	Missing	1	< 1	1	< 1
	Less than high school	108	23	132	21
	High school	91	19	140	23
	Some post-secondary	150	32	248	40
	Graduated university	117	25	99	16
					
Adiposity Category	Least risk	130	28	263	42
	Increased risk	182	39	136	22
	High risk	155	33	221	36

*may not equal 100% due to rounding

**Table 4:** Distribution of lifestyle characteristics in the OFS

		Men N = 467		Women N = 620	
		n	%*	n	%*

LTPA	Missing	9	2	6	< 1
	Inactive	164	35	213	34
	Low	92	20	131	21
	Medium	105	22	141	23
	High	97	21	129	21
					
Smoking status	Missing	1	< 1	1	< 1
	Never	167	36	268	43
	Former	186	40	183	30
	Current	113	24	168	27
					
Daily servings of fruit	Missing	7	2	8	1
	Less than1	55	12	84	14
	1 to 2.9	188	40	240	39
	3 to 4.9	123	26	178	29
	5 or more	94	20	110	18
					
Daily servings of vegetables	Missing	11	2	4	< 1
	Less than1	14	3	33	5
	1 to 2.9	177	38	266	43
	3 to 4.9	153	33	186	30
	5 or more	112	24	131	21

*may not equal 100% due to rounding

### Men

**Figure 2:**
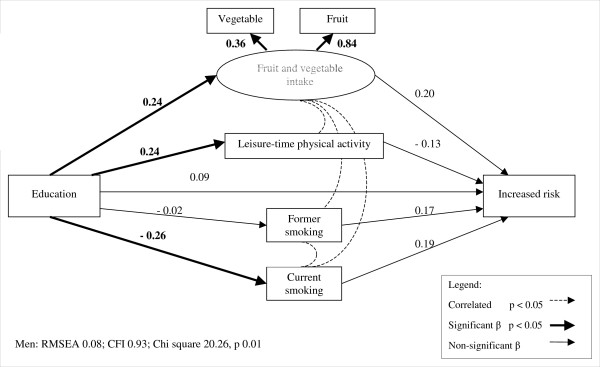
**Structural equation model of increased risk adiposity among men in relation to education and lifestyle factors. **Note: parameter values have been standardized.

**Figure 3:**
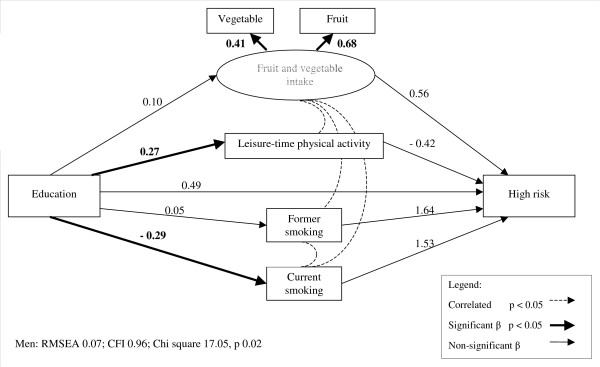
**Structural equation model of high risk adiposity among men in relation to education and lifestyle factors. **Note: parameter values have been standardized.

In all models for men, income and education were positively related to fruit and vegetable intake and LTPA and negatively related to current smoking status (figures 2 and 3, table 5). The likelihood of being in the increased risk adiposity group was unrelated to income or education directly, and was similarly unrelated to income or education through indirect pathways via fruit and vegetable intake, LTPA, or smoking status. Among men, high risk adiposity was not directly related to education, nor indirectly related through any of the behavioural factors. Similarly, high risk adiposity was not related to income among men, though in this model there was a marginally significant inverse association with LTPA. However, the indirect pathway from income to high risk adiposity via LTPA was found to be not significant (table 6). Overall, income and education were not related to either increased or high risk adiposity, despite significant variation in behavioural factors across income and education levels.

**Table 5:** SEM analyses of the interrelationships among income, lifestyle behaviours, and adiposity risk

Dependent	Independent ^a^	Men ^b^		Women ^c^	
**Increased vs. least risk**		Standardizedβ	P value	Standardizedβ	P value

Increased risk	Income	0.07	0.603	0.01	0.921
	Fruit and vegetable	0.16	0.596	-0.15	0.497
	LTPA	-0.13	0.181	0.00	0.992
	Current smoking	0.11	0.881	-0.48	0.385
	Former smoking	0.09	0.905	-0.27	0.603
					
Fruit and vegetable	Income	0.28	0.002	0.15	0.041
LTPA	Income	0.26	0.0003	0.18	0.005
Current smoking	Income	-0.27	0.003	-0.21	0.008
Former smoking	Income	0.05	0.582	0.22	0.006

		Men ^d^		Women ^e^	

**High vs. least risk**		Standardizedβ	P value	Standardizedβ	P value

High risk	Income	0.19	0.478	-0.22	0.010
	Fruit and vegetable	0.36	0.509	-0.35	0.040
	LTPA	-0.35	0.088	-0.10	0.168
	Current smoking	1.00	0.522	-0.26	0.555
	Former smoking	1.13	0.490	-0.12	0.764
					
Fruit and vegetable	Income	0.24	0.022	0.16	0.023
LTPA	Income	0.29	0.0002	0.14	0.019
Current smoking	Income	-0.31	0.0003	-0.25	0.0005
Former smoking	Income	0.13	0.124	0.15	2.08

^a ^Age included as a covariate with all sets of independent variables, results not shown

^b ^n = 312; CFI 0.94; RMSEA 0.07; Chi-square 17.95, p value 0.01

^c ^n = 399; CFI 0.90; RMSEA 0.08; Chi-square 26.83, p value 0.0004

^d ^n = 285; CFI 0.97; RMSEA 0.06; Chi-square 15.02, p value 0.04

^e ^n = 484; CFI 0.94; RMSEA 0.08; Chi-square 26.67, p value 0.0004

### Women

**Figure 4:**
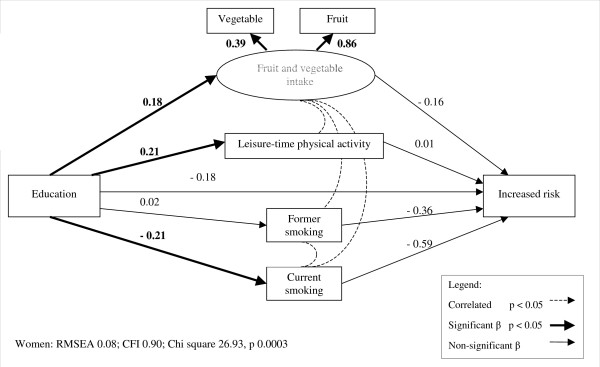
**Structural equation model of increased risk adiposity among women in relation to education and lifestyle factors. **Note: parameter values have been standardized.

**Figure 5:**
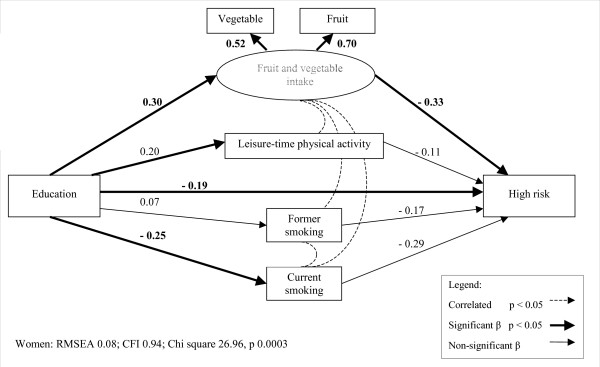
**Structural equation model of high risk adiposity among women in relation to education and lifestyle factors. **Note: parameter values have been standardized.

As seen with men, income and education were positively associated with fruit and vegetable intake and LTPA and negatively associated with current smoking in all models for women (figures 4 and 5, table 5). Similarly, the likelihood of increased risk adiposity was not related to income or education among women, either directly or indirectly via behavioural factors. However, the models for high risk adiposity yielded results that were distinct from those seen with men. High risk adiposity was inversely associated with income and education among women, and also with fruit and vegetable intake. Therefore, indirect pathways were present from both income and education to high risk adiposity via fruit and vegetable intake, given the positive association of fruit and vegetable intake with both income and education. The indirect pathway from education to high risk adiposity via fruit and vegetable intake approached significance, but the corresponding coefficient from the income model was not significant (table 6).

**Table 6:** Summary of indirect pathways from socioeconomic indicators to adiposity groups through lifestyle factors

			Men		Women	
Independent	Mediator	Dependent	Indirect β	P value	Indirect β	P value

Income	Fruit and vegetable	Increased risk	0.05	0.603	-0.02	0.516
	LTPA	Increased risk	-0.03	0.201	0.00	0.999
	Current smoking	Increased risk	-0.03	0.881	0.10	0.407
	Former smoking	Increased risk	0.004	0.905	-0.06	0.610
Education	Fruit and vegetable	Increased risk	0.05	0.624	-0.03	0.509
	LTPA	Increased risk	-0.03	0.246	0.003	0.881
	Current smoking	Increased risk	-0.05	0.849	0.12	0.407
	Former smoking	Increased risk	-0.003	0.889	-0.01	0.842
Income	Fruit and vegetable	High risk	0.09	0.529	-0.06	0.134
	LTPA	High risk	-0.10	0.114	-0.01	0.212
	Current smoking	High risk	-0.31	0.535	0.06	0.576
	Former smoking	High risk	0.15	0.503	-0.02	0.764
Education	Fruit and vegetable	High risk	0.05	0.667	-0.10	0.071
	LTPA	High risk	-0.11	0.396	-0.02	0.142
	Current smoking	High risk	-0.44	0.675	0.07	0.555
	Former smoking	High risk	0.08	0.697	-0.01	0.711

## Discussion

For women in the present analyses, education and income were inversely related to high risk adiposity, but not increased risk adiposity. Neither education nor income was associated with either level of adiposity risk among men, despite the fact the associations of income and education with lifestyle factors were parallel between the sexes. The observation that socioeconomic variation in adiposity occurred only in women is consistent with previous studies [1,3,4], and suggests that the norms of acceptable weight within socioeconomic groups may be more strongly established for women. Previous research has found that dietary restriction is more likely among women with higher family income than those with low family income [49]; similarly, women of higher socioeconomic status have indicated that they are more likely to make dietary choices based on concerns for health or weight status than their counterparts in lower SES groups [50]. Men and women in the OFS both reported greater fruit and vegetable intake with increasing levels of income or education; however, fruit and vegetable intake was associated with a decreased likelihood of high risk adiposity only among women. Therefore, higher fruit and vegetable intake may have been an indicator of a diet geared towards health and/or a lower body weight among women in the OFS, but not among men.

For women, fruit and vegetable intake was a marginally significant mediator of the association between education and high risk adiposity, but was not a significant mediator between income and high risk adiposity despite having significant independent associations with both factors. In the OFS, income was measured at household level whereas education was measured at the level of the individual; therefore, the standard error may have been relatively smaller when testing for indirect associations from education to other individual-level variables (e.g. fruit and vegetable intake or adiposity) compared to indirect associations from income. Also, other studies have indicated that some of the positive association of income and education with fruit and vegetable intake occurs through different mechanisms. Education has been associated with increased nutritional knowledge [51,52], increased social support for fruit and vegetable intake [51], and consideration for health [51]. Income, on the other hand, has been found to impact diet through concerns over food cost and through the purchase of foods that are consistent with dietary recommendations for healthy eating [52]. Within the OFS, education appears to have a greater influence on fruit and vegetable intake than income, yielding a marginally significant indirect pathway to high risk adiposity.

Many of the associations observed with lifestyle factors in the present analyses were consistent with the literature: the socioeconomic indicators have previously been positively associated with fruit and vegetable intake and LTPA [1,2,14,19-21,23-27], and negatively associated with current smoking [1,2,19,20]. In many ways, though, it is the absence of expected associations in the present analyses that is of most interest. The interrelationships of income, education, and lifestyle factors were not significantly different between the increased risk group and the least risk group. Also, smoking status was not associated with increased or high risk adiposity in any of the models; the literature review that led to the development of the models had indicated that adiposity might be associated with smoking status in our sample. The lifestyle factors under examination in the present study follow a clear and consistent socioeconomic pattern; the multivariate approach allowed for the identification of which lifestyle factors were subsequently related to adiposity, independent of each other. These results highlight the need to conduct multivariate analyses that incorporate several factors previously linked to the outcome variable of interest.

The interrelationships observed with lifestyle factors and socioeconomic indicators were not the same between models of increased risk and high risk adiposity among women in the OFS. More specifically, the present analyses indicated that income and education were not predictive of increased risk adiposity compared to least risk adiposity, despite presenting patterns of fruit and vegetable intake, LTPA, and smoking that were similar to those seen in the models of high risk adiposity for women. The dissimilarities between increased risk and high risk adiposity in the OFS require further study to more fully understand the differences between these adiposity levels in relation to socioeconomic factors.

A priori assumptions of presumed directional associations can be tested in SEM but, given the cross-sectional nature of the OFS, causal relationships cannot be established. The low response rate of the OFS prohibits drawing conclusions at the population level. The analysis of non-response bias indicated that participants in the OFS were more likely to be highly educated than the population in general [42]; therefore, the extent to which high education could be contrasted against low education was somewhat stunted in the present analyses, and may have resulted in an underestimation of the true association. The dietary practices and leisure-time activities reported at the time of the survey may not represent habits over time, particularly if participants were modifying their behaviour in an effort to lose weight. Specifically, higher levels of LTPA and fruit and vegetable intake in the period preceding the survey may, in part, explain the absence of findings among adults with increased risk adiposity or men in the high risk adiposity group. With the exception of measured variables for adiposity, which were obtained from 75% of the sample, the present analyses were reliant upon self-reported values, and the potential for error must be acknowledged. In addition, the measurement properties of the instruments used to collect data on LTPA and fruit and vegetable intake are unknown. However, many of the relationships observed in the present analyses had been previously detected in other populations with validated instruments [14,30,53,54], suggesting that the quality of the OFS data was reasonable.

Not all of the variables that are potentially relevant for the study of socioeconomic variation in adiposity were available in the OFS. It has been proposed that lower occupational status is associated with high levels of stress and restricted time for food preparation and physical activity, and conversely that people in higher status occupations are more concerned about weight status and more likely to be dieting [6]. In addition, data on reproductive history might have improved the present analyses among women because of the increased parity among women of lower socio-economic status, and the weight gain associated with childbearing [55]. One study found that reproductive history accounted for more of the associations observed for a composite measure of socioeconomic status with overweight and obesity than either diet or psychosocial stress alone (44% vs. 40% and 33% respectively)[55]. Finally, social desirability has been associated with dietary reporting among women and could have refined the present analyses [56], particularly if social desirability were positively associated with socioeconomic indicators. The inclusion of these factors in future analyses may provide further insight into socioeconomic variation in adiposity.

## Conclusion

Women with low income and low education were particularly vulnerable to high risk adiposity in the OFS, despite a comparable prevalence of high risk adiposity among men and women. The results of the present study must be replicated before the relative contributions of LTPA, smoking, and fruit and vegetable intake to this inverse association can be firmly established. However, the present research has served to reinforce that the socioeconomic context of adiposity is distinct for men and women, and consideration for these differences should be incorporated into population health intervention efforts to reduce the prevalence of high risk adiposity.

## Competing interests

The author(s) declare that they have no competing interests.

## Authors' contributions

HW carried out the statistical analyses and drafted the manuscript. VT made substantial contributions to the conception of the study, critically revised the manuscript, and was a co-investigator of the Ontario Food Survey. RM was the Principal Investigator of the Ontario Food Survey, participated in the design of the study, and provided critical feedback on the manuscript. GME made substantial contributions to the analytic approach and interpretation of the data. All authors read and approved the final manuscript.
